# Correction to: The Role of TIR-NBS and TIR-X Proteins in Plant Basal Defense Responses

**DOI:** 10.1093/plphys/kiac323

**Published:** 2022-07-20

This is a correction to: Raja Sekhar Nandety, Jeffery L. Caplan, Keri Cavanaugh, Bertrand Perroud, Tadeusz Wroblewski, Richard W. Michelmore, Blake C. Meyers, The Role of TIR-NBS and TIR-X Proteins in Plant Basal Defense Responses, Plant Physiology, Volume 162, Issue 3, July 2013, Pages 1459–1472, https://doi.org/10.1104/pp.113.219162

The authors were made aware of the image duplicated in Figure 6A (p. 1468). In the original version of Figure 6A, the image is identical to one presented in Supplemental Figure 5A. During the preparation of the manuscript, due to the similarity of the localization patterns in the cells and cell shapes, the image duplication went unnoticed. The original, intended image was recovered. The legend of Figure 6 remains unchanged. Below are the original and the corrected versions of Figure 6A.

This correction does not otherwise affect the results nor alter any of the original conclusions. We apologize for any inconvenience to the readers.

**Figure 6.**
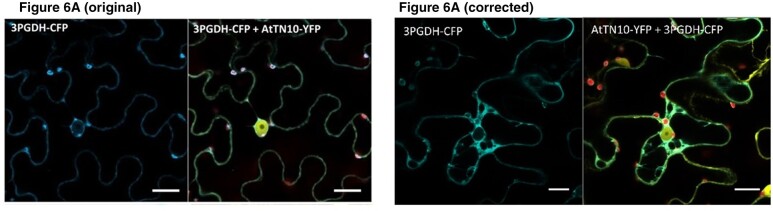
Arabidopsis TN protein AtTN10 interacts with 3PGDH in the cytoplasm. **(A)** AtTN10 transiently co-overexpressed with 3PGDH in tobacco leaves results in cytoplasmic localization. Bars = 20 µm.

